# Association between *ACE* I/D genetic polymorphism and the severity of coronary artery disease in Vietnamese patients with acute myocardial infarction

**DOI:** 10.3389/fcvm.2023.1091612

**Published:** 2023-05-03

**Authors:** Duy Cong Tran, Linh Hoang Gia Le, Truc Thanh Thai, Sy Van Hoang, Minh Duc Do, Binh Quang Truong

**Affiliations:** ^1^ Department of Internal Medicine, University of Medicine and Pharmacy at Ho Chi Minh City, Ho Chi Minh City, Vietnam; ^2^ Department of Cardiology, Cho Ray Hospital, Ho Chi Minh City, Vietnam; ^3^ Cardiovascular Center, University Medical Center Ho Chi Minh City, Ho Chi Minh City, Vietnam; ^4^ Center for Molecular Biomedicine, University of Medicine and Pharmacy at Ho Chi Minh City, Ho Chi Minh City, Vietnam; ^5^ Faculty of Public Health, University of Medicine and Pharmacy at Ho Chi Minh City, Ho Chi Minh City, Vietnam

**Keywords:** genetic polymorphism, coronary artery disease, Gensini score, acute myocardial infarction, Vietnamese, *ACE* I/D

## Abstract

**Background:**

The severity of coronary artery disease is a prognostic factor for major adverse cardiovascular events in patients diagnosed with acute myocardial infarction. *ACE* I/D polymorphism is one of the genetic factors that may affect the severity of coronary artery disease. This study aimed to investigate the association between *ACE* I/D genotypes and the severity of coronary artery disease in patients with acute myocardial infarction.

**Materials and methods:**

A single-center, prospective, observational study was conducted at the Department of Cardiology and Department of Interventional Cardiology, Cho Ray Hospital, Ho Chi Minh City, Vietnam from January 2020 to June 2021. All participants diagnosed with acute myocardial infarction underwent contrast-enhanced coronary angiography. The severity of coronary artery disease was determined by Gensini score. *ACE* I/D genotypes were identified in all subjects by using the polymerase chain reaction method.

**Results:**

A total of 522 patients diagnosed with first acute myocardial infarction were recruited. The patients' median Gensini score was 34.3. The II, ID, and DD genotype rates of *ACE* I/D polymorphism were 48.9%, 36.4%, and 14.7%, respectively. After adjusting for confounding factors, multivariable linear regression analysis showed that the *ACE* DD genotype was independently associated with a higher Gensini score compared with the II or ID genotypes.

**Conclusion:**

The DD genotype of the *ACE* I/D polymorphism was associated with the severity of coronary artery disease in Vietnamese patients diagnosed with first acute myocardial infarction.

## Introduction

Despite major advances in diagnosis and management, acute myocardial infarction (AMI) remains a serious healthcare burden worldwide, significantly increasing patients’ morbidity and mortality (1). The severity of coronary artery disease (CAD) is a prognostic factor for major adverse cardiovascular outcomes in patients with AMI (2–4). Several scoring systems are available for the quantitative evaluation of coronary artery lesions, and among them, the Gensini score is the most frequently used in clinical settings. The Gensini score assesses the quantity, location, and degree of stenosis of epicardial coronary artery lesions, providing a scientific evaluation standard for CAD severity (5).

In addition to environmental factors, genetic components have been revealed to be associated with the severity of CAD in AMI patients. Recent research has shown that CAD severity is influenced by variations in the angiotensin-converting enzyme (*ACE*) gene (6, 7). ACE, an important component of the renin-angiotensin-aldosterone system, converts angiotensin I to angiotensin II and inactivates bradykinin *via* the kallikrein-kininogen system (8). The *ACE* gene is located on the long arm of chromosome 17 (17q23), has a length of 21 kilobases (kb), and contains 26 exons and 25 introns. Insertion (I) and deletion (D) polymorphisms in the intron 16 of the *ACE* gene are defined by the presence or absence of a 287 bp Alu repeat (9). High serum ACE levels vary in the order homozygote deletion (DD) > heterozygote (ID) > homozygote insertion (II) (9). Cardiac ACE activity is also higher in DD-carrying subjects (10). High ACE levels are associated with an increased angiotensin II concentration, with harmful effects including vasoconstriction, aldosterone secretion, cellular proliferation, vascular remodeling, and oxidative stress; these effects give rise to endothelial dysfunction and atherosclerosis (8).

Since it was first described in the 1990s, the *ACE* I/D genetic polymorphism has been shown to be a potential risk factor for AMI. Cambien and colleagues were the first to report an association between the *ACE* I/D genetic variant and the risk of AMI; the study sample from ECTIM (Etude Cas-Temoin de l'Infarctus du Myocarde) included 610 men aged 25–64 years who had survived for 3–9 months after AMI and 733 participants with no history of CAD (11). The association between *ACE* I/D polymorphism and the risk of AMI has been studied further, but the findings are inconsistent (12–14). A meta-analysis by Chen et al. concluded that the D allele of the *ACE* I/D genetic polymorphism is a potential risk factor for AMI in both Asians and Caucasians (15).

Although *ACE* I/D genetic variants have been extensively studied in relation to AMI, only a few studies have investigated the clinical significance of *ACE* I/D genotypes to the severity of CAD. In addition, the severity of CAD in the previous studies was mostly based on the number of stenosed coronary arteries, rather than the Gensini score. Thus, there is a lack of data on the association between *ACE* I/D genetic polymorphism and CAD severity evaluated using the Gensini score. The identification of factors affecting the severity of CAD can contribute to strategies for the primary and secondary prevention of AMI. Therefore, this study aimed to investigate the association between *ACE* I/D genotypes and the severity of CAD assessed using the Gensini score for Vietnamese patients with AMI.

## Materials and methods

### Study design and population

This was a single-center, prospective, observational study conducted at the Department of Cardiology and Department of Interventional Cardiology, Cho Ray Hospital, Ho Chi Minh City, Vietnam from January 2020 to June 2021. This research was approved by the Ethics Committee in Biomedical Research of the University of Medicine and Pharmacy at Ho Chi Minh City (HEC/IRB number 550/UMP-BOARD). Eligible patients were ≥18 years old with a confirmed diagnosis of AMI according to the fourth universal definition from the European Heart Association, the American College of Cardiology, the American Heart Association, and the World Heart Federation published in 2018 (16). Patients who had any of the following criteria were excluded: (1) a history of myocardial infarction, percutaneous coronary intervention, or coronary artery bypass surgery; (2) no coronary angiography performed; (3) no stenosis or less than 50% luminal diameter stenosis of all major epicardial coronary arteries; (4) clinical presentations of connective tissue diseases such as systemic lupus erythematosus, rheumatoid arthritis, scleroderma, systemic vasculitis, antiphospholipid syndrome, amyloidosis, thyroid diseases, and active cancer; (5) refused to provide the informed consent or did not want to take part in this study.

Demographic data and data on cardiovascular risk factors, prior use of medications, clinical type of AMI, Killip class, lipid profile, and estimated glomerular filtration rate (eGFR) were collected. Obesity was defined as a body mass index (BMI) ≥ 25 kg/m^2^ for the Asia-Pacific population (17). BMI was calculated as weight (kg) divided by the square of height (m) (18); body surface area was estimated by the square root of the height (cm) multiplied by the weight (kg) divided by 3,600 (19). Hypertension was defined as either a previous diagnosis of hypertension or newly-diagnosed hypertension with at least two times of blood pressure measurements ≥140/90 mmHg according to the 2018 European Society of Hypertension and the European Heart Association guidelines (20). A prior or new diagnosis of diabetes was defined according to the diagnostic criteria of American Diabetes Association (21). Smoking was defined as a patient who was currently smoking or had stopped smoking within the last 12 months (22). Dyslipidemia was defined as having one of the following abnormalities: total cholesterol ≥200 mg/dl, high-density lipoprotein (HDL) cholesterol <40 mg/dl, low-density lipoprotein (LDL) cholesterol ≥130 mg/dl, triglyceride ≥150 mg/dl, or a previous diagnosis of dyslipidemia (23). A family history of premature CAD was defined when there were any first-degree male relative <55 years old or a female relative <65 years old having CAD (24). ST-segment elevation myocardial infarction (STEMI) was confirmed if AMI patients had new ST-elevation at the J-point in two contiguous leads with a cut-off point of ≥1 mm in all leads other than leads V2-V3, where the following cut-off points applied: ≥ 2 mm in men ≥40 years, ≥ 2.5 mm in men <40 years, and ≥1.5 mm in women regardless of age (16). Non-ST-segment elevation myocardial infarction (NSTEMI) was confirmed if AMI patients had new horizontal or downsloping ST-depression of ≥0.5 mm in two contiguous leads and/or T inversion >1 mm in two contiguous leads with a prominent R wave or an R/S ratio >1 (16).

### Assessment of coronary angiography

All participants underwent contrast-enhanced coronary angiography, and at least two interventional physicians assessed the results. Significant lesions of coronary arteries were defined as ≥50% stenosed diameter on coronary angiography. The Gensini score was applied by two experienced cardiologists to determine the severity of CAD (5). When Gensini scores were not consistent between the two assessors, they discussed with each other and decided the final exact result. The degree of stenosis and the location of the coronary artery lesions were scored using the following scale: 1 point for less than 25% stenosis, 2 points for 26%–50% stenosis, 4 points for 51%–75% stenosis, 8 points for 76%–90% stenosis, 16 points for 91%–99% stenosis, and 32 points for total occlusion (5).

Each lesion score was then multiplied by a factor that took into consideration the importance of the lesion's site in the coronary tree (5 for the left main coronary artery, 2.5 for the proximal segment of the left anterior descending artery, 2.5 for the proximal segment of the circumflex artery, 1.5 for the mid-segment of the left anterior descending artery, 1.0 for the right coronary artery, the distal segment of the left anterior descending artery, the posterolateral artery, and the obtuse marginal artery, and 0.5 for other segments). The sum of the scores from each coronary segment was used to calculate the Gensini score (5).

### Genetic analysis

The *ACE* I/D genotypes were identified by the polymerase chain reaction (PCR) method at the Center for Molecular Biomedicine, University of Medicine and Pharmacy at Ho Chi Minh City, Vietnam. A sample of 2 milliliters (ml) of venous blood was taken from each patient, placed in a tube anticoagulated with EDTA, and gently shaken. Genomic DNA from white blood cells was extracted within 24 h using the GeneJet TM Whole Blood Genomic DNA Purification Mini Kit (Thermo Fisher Scientific, Waltham, MA, USA) according to the manufacturer's instructions. Primers for PCR were designed using CLC Main Workbench software based on the human *ACE* gene sequence (Genebank NG_011648).

The conditions for PCR amplification to create sequence regions were that each PCR tube had a volume of 15 microliters (µl) containing the following components: 1.5 μl PCR buffer 10X; 1.5 μl dNTP 2.5 mM; 0.75 μl each forward and reverse primer (10 nM/μl), 0.1 μl TaKaRa TaqTM HotStart Polymerase (Takara, Japan), 2 μl genomic DNA (20–50 ng/μl), and 8.4 μl double distilled deionized water. PCR responses were accompanied by a negative control that did not contain DNA for external infection control and positive controls that were variants of the *ACE* I/D previously identified by the Sanger DNA sequencing. Thermal cycles for PCR were performed using a Mastercycler@Pro S (Eppendorf, Hamburg, Germany). The thermal cycles consisted of one cycle at 98°C for 3 min and 40 cycles of denaturation at 98°C for 10 s, annealing at 60°C for 20 s, extension at 72°C for 30 s, and final extension at 72°C for 2 min. PCR products were stored at 4°C and identified by electrophoresis on 2% agarose agar, stained with Gel Red, and observed using a Geldoc-ItTM electrophoresis imaging system (UVP, USA). A single band of 510 bp was observed for the II genotype and 206 bp for the DD genotype; both bands were observed for the heterozygous ID genotype. The protocol for *ACE* I/D genotyping is described in Supplementary Material, Table S1. To assure the genotyping accuracy, 10% of the samples were randomly chosen and directly sequenced using the previously described protocol with appropriate primers (25, 26).

### Data analysis

Data were analyzed using SPSS 22.0 for Windows software (SPSS, Inc). Continuous variables are presented as mean (standard deviation) or median (interquartile range) when depending on data distribution. Categorical variables were presented in the form of frequency and percentages. Differences in characteristics between three *ACE* I/D genotypes were assessed using Chi-squared or Fisher's exact tests for categorical variables and ANOVA or Kruskal Wallis tests for continuous variables. Differences in Gensini scores between *ACE* I/D genotypes in the genetic models (the recessive model: DD vs. II + ID, the dominant model: II vs. ID + DD, the homozygous model: DD vs. II, and the heterozygous model: ID vs. II) were compared using the Student's t-tests or the Mann-Whitney *U* tests.

The association between participant's characteristic and the severity of CAD in AMI were investigated first by univariate linear regression analysis. Multivariable linear regression model was then fitted using all significant variables in univariate analysis. A *p*-value of < 0.05 was considered statistically significant.

## Results

During the study period, 522 AMI patients were recruited. The baseline characteristics of participants are presented in Table 1. The mean age of subjects was 63.9 ± 11.7. Males were predominant (71.5%). Dyslipidemia and hypertension were the most common cardiovascular risk factors. The percentages of *ACE* II, ID, and DD genotypes were 48.9%, 36.4%, and 14.7%, respectively. There was no significant difference in clinical and laboratory parameters between *ACE* I/D genetic polymorphism categories.

**Table 1:** Baseline characteristics of participants by *ACE* I/D genotype.

Characteristics	Total (*n* = 522)	II (*n* = 255, 48.9%)	ID (*n* = 190, 36.4%)	DD (*n* = 77, 14.7%)	*P*-value
Age (years) (Mean ± SD)	63.9 ± 11.7	64.3 ± 12.1	63.4 ± 11.0	64.0 ± 12.2	0.703
Male (*n*, %)	373 (71.5)	186 (72.9)	132 (69.5)	55 (71.4)	0.725
Obesity (*n*, %)	107 (20.5)	51 (20.0)	42 (22.1)	14 (18.2)	0.743
Hypertension (*n*, %)	426 (81.6)	203 (79.6)	159 (83.7)	64 (83.1)	0.511
Diabetes mellitus (*n*, %)	128 (24.5)	59 (23.1)	48 (25.3)	21 (27.3)	0.728
Smoking (*n*, %)	222 (42.5)	115 (45.1)	80 (42.1)	27 (35.1)	0.293
Dyslipidemia (*n*, %)	467 (89.5)	225 (88.2)	169 (88.9)	73 (94.8)	0.248
Prior use of ACEi/ARB (*n*, %)	364 (66.3)	169 (66.3)	123 (64.7)	54 (70.1)	0.700
Prior use of beta-blocker (*n*, %)	159 (30.5)	83 (32.5)	58 (30.0)	19 (24.7)	0.415
Prior use of MRA (*n*, %)	18 (3.4)	11 (4.3)	5 (2.6)	2 (2.6)	0.571
Prior use of antiplatelets (*n*, %)	56 (10.7)	24 (9.4)	21 (11.1)	11 (14.3)	0.472
Prior use of statins (*n*, %)	274 (52.5)	134 (52.5)	101 (53.2)	39 (50.6)	0.933
Total cholesterol (mg/dl) (Median [IQR])	174.0 (147.0–205.0)	176.0 (146.0–204.0)	173.0 (147.8–206.0)	172.0 (149.0–205.5)	0.754
LDL cholesterol (mg/dl) (Median [IQR])	122.0 (92.0–150.0)	119.0 (88.0–150.0)	125.0 (95.8–152.5)	125.0 (100.0–151.0)	0.349
HDL cholesterol (mg/dl) (Median [IQR])	35.0 (30.0–42.0)	35.0 (30.0–43.0)	35.0 (30.0–41.0)	35.0 (30.0–39.5)	0.629
Triglyceride (mg/dl) (Median [IQR])	146.0 (106.0–210.0)	153.0 (104.0–208.0)	155.0 (109.0–222.3)	136.0 (110.0–189.5)	0.398
eGFR (ml/min/1.73 m^2^) (Median [IQR])	82.9 (64.9–94.5)	83.5 (67.4–94.0)	84.2 (66.6–95.3)	75.1 (59.8–95.2)	0.234

SD, standard deviation; ACEi, angiotensin-converting enzyme inhibitor; ARB, angiotensin II receptor blocker; MRA, mineralocorticoid receptor antagonist; IQR, interquartile range; eGFR, estimated glomerular filtration rate; LDL, low-density lipoprotein; HDL, high-density lipoprotein.

The clinical type of AMI, Killip class and coronary angiographic features are shown in Table 2. STEMI occurred in 64.4% of patients, the majority of whom exhibited Killip class I (76.4%). The percentage of multivessel disease was 75.5%. There was no significant difference in the location and number of diseased coronary arteries between *ACE* I/D genotypes.

**Table 2:** Clinical diagnosis and angiographic characteristics by *ACE* I/D genotype.

Characteristics	Total (*n* = 522)	II (*n* = 255)	ID (*n* = 190)	DD (*n* = 77)	*P*-value
**Clinical type of AMI**					
STEMI (*n*, %)	336 (64.4)	163 (63.9)	126 (66.3)	47 (61.0)	0.702
NSTEMI (*n*, %)	186 (35.6)	92 (36.1)	64 (33.7)	30 (39.0)	
**Killip class**					
Class I (*n*, %)	399 (76.4)	196 (67.9)	143 (75.3)	60 (77.9)	0.823
Class II (*n*, %)	44 (8.4)	23 (9.0)	17 (8.9)	4 (45.2)	
Class III (*n*, %)	32 (6.1)	17 (6.7)	10 (5.3)	5 (6.5)	
Class IV (*n*, %)	47 (9.1)	19 (7.5)	20 (10.5)	8 (10.4)	
**Location of diseased coronary arteries**					
LM (*n*, %)	52 (10.0)	25 (9.8)	20 (10.5)	7 (9.1)	0.933
LAD (*n*, %)	645 (89.1)	226 (88.6)	170 (89.5)	69 (89.6)	0.948
LCx (*n*, %)	290 (55.6)	148 (58.0)	99 (52.1)	43 (55.8)	0.459
RCA (*n*, %)	371 (71.1)	174 (68.2)	144 (75.8)	53 (68.8)	0.198
**Number of diseased coronary arteries**					
1 (*n*, %)	128 (24.5)	64 (25.1)	45 (23.7)	19 (24.7)	0.966
2 (*n*, %)	184 (35.2)	89 (34.9)	67 (35.3)	28 (36.4)	
3 (*n*, %)	210 (40.2)	102 (40.0)	78 (41.1)	30 (30.9)	
Multivessel disease (%)	394 (75.5)	191 (74.9)	145 (76.3)	58 (75.3)	0.942

AMI, acute myocardial infarction; STEMI, ST-segment elevation myocardial infarction; NSTEMI, non-ST-segment elevation myocardial infarction; LM, left main; LAD, left anterior descending artery; LCx, left circumflex artery; RCA, right coronary artery.

The median Gensini score was 34.3 (IQR = 17.0–58.3). The *ACE* DD genotype was associated with the severity of CAD evaluated by the Gensini score in the recessive model (DD vs. II + ID) (Table 3). Patients with DD genotype had higher Gensini scores than those with ID or II genotypes (*p* = 0.046). This association was not found in the other genetic models including the dominant, homozygous, and heterozygous models.

**Table 3:** Association of *ACE* I/D genotypes with Gensini scores.

Genetic model	*n*	Gensini scores	*P*-value
**Recessive model (DD vs. II + ID)**			
DD	77	38.0 (20.0–73.0)	0.046*
II + ID	445	34.0 (17.0–56.0)	
**Dominant model (II vs. ID + DD)**			
II	255	34.0 (18.0–60.0)	0.751
ID + DD	267	34.5 (17.0–57.0)	
**Homozygous model (DD vs. II)**			
DD	77	38.0 (20.0–73.0)	0.119
II	255	34.0 (18.0–60.0)	
**Heterozygous model (ID vs. II)**			
ID	190	34.0 (16.0–52.3)	0.206
II	255	34.0 (18.0–60.0)	

* Statistically significant.

In terms of factors affecting the severity of CAD in AMI patients, univariate linear regression analysis showed that the *ACE* I/D genetic variant, Killip class, and triglyceride concentration were statistically associated with the Gensini score (Table 4). In multivariable linear regression, *ACE* I/D genetic polymorphism remained statistically associated with the Gensini score. DD-carrying participants presented higher Gensini scores compared with II or ID-carrying subjects (Table 5).

**Table 4:** Univariate linear regression analysis of Gensini scores.

Factors	*β*	95% CI	*P*-value
Age	0.153	−0.076–0.382	0.190
Gender (male vs. female)	−1.587	−7.518–4.344	0.599
Obesity	−2.761	−9.394–3.871	0.414
Hypertension	3.621	−3.288–10.529	0.304
Diabetes mellitus	4.997	−1.216–11.210	0.115
Smoking	−4.461	−9.867–0.945	0.106
Dyslipidemia	6.414	−2.295–15.123	0.149
Family history of premature CAD	−4.398	−15.248–6.453	0.426
Prior use of ACEi/ARB	−0.792	−6.459–4.874	0.784
Prior use of beta-blocker	−3.755	−9.567–2.508	0.205
Prior use of MRA	2.618	−12.063–17.300	0.726
Prior use of antiplatelet	2.496	−6.159–11.151	0.571
Prior use of statin	−1.363	−6.727–4.001	0.618
Clinical type of AMI (STEMI vs. NTEMI)	−1.753	−7.345–3.840	0.538
Killip class	3.314	0.524–6.105	0.020*
Total cholesterol (mg/dl)	0.003	−0.050–0.056	0.923
LDL Cholesterol (mg/dl)	0.042	−0.018–0.102	0.172
HDL Cholesterol (mg/dl)	−0.221	−0.522–0.080	0.149
Triglyceride (mg/dl)	−0.014	−0.028–0.000	0.048*
eGFR (ml/min/1.73 m^2^)	−4.912	−10.450–0.626	0.082
*ACE* DD (vs. II + ID)	8.210	0.688–15.732	0.032*

CAD, coronary artery disease; ACEi, angiotensin-converting enzyme inhibitor; ARB, angiotensin II receptor blocker; AMI, acute myocardial infarction; MRA, mineralocorticoid receptor antagonist; LDL, low-density lipoprotein; HDL, high-density lipoprotein; eGFR: estimated glomerular filtration rate.

* Statistically significant.

**Table 5:** Multivariable linear regression analysis of Gensini scores.

Factors	β	95% CI	*P*-value
*ACE* DD (vs. II + ID)	7.742	0.251–15.233	0.043*
Killip class	3.135	0.353–5.917	0.027*
Triglyceride (mg/dl)	−0.012	−0.026–0.002	0.087

CI, confidence interval.

* Statistically significant.

## Discussion

In this study, patients with AMI had the lowest percentage of the DD genotype (14.7%) compared to the II (48.9%) and ID (36.4%) genotypes. Studies in other Asian countries also found the *ACE* DD genotype to account for the lowest proportion in Japanese, Chinese, and Indian (27–29). In contrast, studies of Western and African populations demonstrated that the II genotype has the lowest proportion (11, 30, 31). Despite differences in the distribution of *ACE* I/D genotypes, several studies indicated that the D allele and DD genotype were associated with the risk of AMI and CAD in both Asians and Caucasians (15, 32).

The Gensini score is frequently used to determine the severity of CAD based on coronary angiography (4, 5, 33). In our study, *ACE* I/D and Killip class were found to be associated with the severity of CAD assessed by Gensini score in patients diagnosed with their first AMI. Factors that were previously reported as associated factors of the severity of CAD (age, gender, cardiovascular risk factors, clinical type of AMI and prior use of ACE inhibitors, angiotensin II receptor blockers, mineralocorticoid receptor antagonists, beta-blockers, antiplatelets, and statins) were not statistically different between *ACE* I/D genotypes. The Gensini score was found to be significantly higher in DD compared with II or ID-carrying participants after adjustment for confounding factors, such as serum triglyceride concentration and Killip class. Chen et al. showed that acute coronary syndrome patients with the DD genotype have a 3.87-fold increased risk of stenosis in three coronary vessels, a 3.08-fold higher risk of stenosis of the left anterior descending artery, and a 3.07-fold higher risk of anterior wall infarction (6). Another study also indicated that the D allele was associated with the number of stenosed coronary arteries, especially its interaction with cardiovascular risk factors, such as sex (male), age (>65 years old), hypertension, diabetes, obesity, and smoking (7). In a group of 647 patients who underwent elective coronary angiography, Borzyszkowska et al. found a significant association between the *ACE* DD genotype and the Gensini score in men with high total cholesterol, high LDL cholesterol, and low HDL cholesterol levels (34). These consistent data emphasize the role of *ACE* I/D as a potential marker for the severity of coronary lesions in patients diagnosed with AMI beside traditional cardiovascular risk factors.

Like other Asian populations, the mechanism of coronary artery disease, especially acute myocardial infarction, in Vietnamese patients might be caused by both traditional cardiovascular risks and genetic factors (35, 36). The observed associations of *ACE* I/D with the severity of coronary artery lesions might be explained by the fact that *ACE* I/D polymorphism is responsible for 20%-50% of the variability in serum ACE level in the following order: DD > ID > II (37). A high concentration of ACE leads to increased synthesis of angiotensin II and the inactivation of bradykinin, which results in increased vascular resistance and hypertension (38). In addition, angiotensin II regulates the proliferation, migration, and hypertrophy of vascular smooth muscle cells and therefore plays a crucial role in the pathophysiology of coronary atherosclerosis (39).

There were several limitations to this study. First, our study is from a single center, and thus may not represent the characteristics of AMI and *ACE* I/D genetic polymorphism in the wider Vietnamese population. Second, the Gensini score was chosen to evaluate the severity of CAD in AMI, which did not consider bifurcation, calcification and distortion of coronary artery lesions compared to the SYNTAX score. Nevertheless, the Gensini score has been widely used in studies where significantly positive association between the Gensini score with the GRACE score and prognosis in AMI patients were reported (4, 33). Third, the serum ACE concentration was not measured and determined its association with different *ACE* I/D genotypes and the severity of CAD. Finally, as AMI is a polygenic disease, *ACE* I/D may not comprehensively explain the difference in CAD severity between genotype groups. Thus, further research with complete genetic analysis, such as genome-wide association study, is required to better understand the association between genetic factors and the severity of CAD in Vietnamese patients with AMI.

In conclusion, the *ACE* DD genotype was found to be associated with the severity of CAD compared with the II or ID genotypes in Vietnamese patients with their first AMI. To the best of our knowledge, this work is the first study in Vietnam describing the association between a genetic polymorphism and the severity of coronary artery lesion, and it contributes to the literature of the role of *ACE* I/D polymorphism in the pathophysiology of AMI in Asian populations. Therefore, *ACE* I/D should be considered to improve the comprehensive assessment and aggressive treatment of AMI patients.

## Data Availability

The original contributions presented in the study are included in the article/**Supplementary materials**, further inquiries can be directed to the corresponding authors.
