# Supplementary material for: Association between ACE I/D genetic polymorphism and the severity of coronary artery disease in Vietnamese patients with acute myocardial infarction
Source: Front Cardiovasc Med. 2023 May 3;10:1091612. doi: 10.3389/fcvm.2023.1091612 (PMC10188916; doi:10.3389/fcvm.2023.1091612)
Supplement: Supplementary file 1 [file Table1.docx]

**Supplementary Table 1.** Protocol for *ACE* I/D genotyping

| **Primers** | **Primer sequences** | **Length of PCR products (bp)** | **Melting temperature (^0^C)** |
| --- | --- | --- | --- |
| ACE-F | 5’- ACTCTGTAAGCCACTGCTGG -3’ | **II**: 510; **ID**: 206 and 510; **DD**: 206 | 55 |
| ACE-R | 5’- GATGTGGCCATCACATTCGTCA-3’ |  | 57 |

*PCR, polymerase chain reaction; bp, base pair*
